# How Refugees’ Stereotypes Toward Host Society Members Predict Acculturation Orientations: The Role of Perceived Discrimination

**DOI:** 10.3389/fpsyg.2021.612427

**Published:** 2021-02-05

**Authors:** Sebastian Lutterbach, Andreas Beelmann

**Affiliations:** Department of Research Synthesis, Intervention and Evaluation, Friedrich Schiller University, Jena, Germany

**Keywords:** stereotypes, acculturation, shared reality, discrimination, refugees

## Abstract

Refugee migration leads to increased diversity in host societies and refugees have to face many stereotyped attitudes in the host society. However, there has been little research on minority group stereotypes toward host society members and how these stereotypes relate to the acculturation-relevant attitudes of refugees in their first phase of acculturation. This study surveyed 783 refugees in Germany who had migrated mostly in the so-called “refugee crisis” between 2015 and 2016. At the time of the survey in 2018, they had been in Germany for an average of 27 months (*SD* = 15 months). These refugees reported their positive and negative sociability stereotypes toward German host society members, acculturation-related orientations, shared reality values, and perceived discrimination. Results showed that positive sociability stereotypes toward host society members were associated with increased cultural adoption and shared reality. In contrast, negative sociability stereotypes negatively affected cultural adoption and shared reality. However, stereotypes showed no association at all with cultural maintenance. Interactions between sociability stereotypes and discrimination experiences highlighted a disillusion effect, in the sense that discrimination reduced the motivation to adopt the host culture more strongly among refugees who held strongly positive sociability stereotypes. The study extends knowledge on the significance of minority group stereotypes in the context of refugee migration and reveals the maladaptive consequences of discriminatory behavior against refugees by host society members.

## Introduction

Every year, thousands of people across the globe migrate to another country, into another culture, and into another social context searching for ways to improve their lives. Forced refugee migration to Europe increased since 2011 all the way up to the so-called “refugee crisis” in 2015 and 2016 (Bundesamt für Migration und Flüchtlinge BAMF, 2019). Subsequently, refugees from the Middle East, and North Africa have become the largest group migrating to Germany. These increased numbers of refugees have led to more and intensified contacts with members of the German host society, thus putting the issue of coexistence and acculturation at the heart of public and political debate. Typically, these discussions focus on the acculturative interests and values of the host society and largely ignore refugees’ experiences, their perspectives on integration-relevant attitudes, and the factors that influence minority acculturation orientations. Research on refugee acculturation is dominated by studies on refugees’ physical and psychological well-being (Virgincar et al., 2016; Bas-Sarmiento et al., 2017; Turrini et al., 2017) or on bringing together well-being and acculturation perspectives (Lincoln et al., 2015; LeMaster et al., 2018; Hashemi et al., 2019; Berry and Hou, 2020). However, there has been less research on cognitive variables (such as migration-relevant expectations and beliefs regarding future relations with host society members) and how these influence the acculturation process, contact experiences with the host society, as well as acculturation-relevant beliefs and behaviors of refugees.

Especially stereotypes play a prominent role in shaping cognitive evaluation, beliefs, and behavioral motivations (Worchel, 1999; Fiske et al., 2002), and they have been shown to affect acculturation orientations (López-Rodríguez and Zagefka, 2015; Alcott and Watt, 2017) at least among host society members. However, there is no research addressing the effects of stereotypes on acculturation-relevant beliefs in the case of refugees or immigrants. Due to the mutuality of acculturation (intergroup processes resulting from acculturation and shaping acculturation of the host society and migrants, Berry, 2006; Horenczyk et al., 2013), it is also important to take the quality of intergroup experiences with the cultural outgroup into account, and especially, how discriminatory behavior by the host society affects acculturation of refugees. Consequently, the present study investigated the relationships between positive and negative sociability stereotypes held by refugees toward German host society members (do Germans have good or bad intentions toward refugees) and the motivation to adopt the host culture, maintain one’s own cultural identity, and develop shared reality perceptions (the belief of an in-group member that the own group perceives the world in the same way as a relevant outgroup). Furthermore, we analyzed the role of different types of perceived discrimination on acculturation-relevant perspectives, as well as interactions between stereotypes and discrimination experiences to shed some light on the impact of discrimination experiences in amplifying or reducing the effects of positive and negative stereotypes on acculturation-relevant perspectives.

### Acculturation Orientations and Shared Reality

Within psychology, the most influential contribution to theorizing about the coexistence of minority and majority groups is Berry’s acculturation model (Berry, 1997; Sam and Berry, 2016). The framework of his model focuses on two principal dimensions that underlie immigrants’ acculturation orientations: their desire to maintain their original culture and their desire to have contact with majority group members. Subsequent research has concluded that the contact dimension should be replaced by one highlighting the desire to adopt the norms, values, and cultural behaviors of the host society, because it provides a better match with the cultural maintenance dimension (Bourhis et al., 1997). Both acculturation orientations together give rise to four discrete acculturation strategies (integration, assimilation, separation, and marginalization; see Berry, 1997) with which to differentiate acculturation processes by both majority and minority groups. Integration is defined by the motivation to retain one’s cultural traditions while also adopting cultural aspects of the cultural outgroup, whereas marginalization, in contrast, is specified by the rejection of both cultural identities. Assimilation orientations highlight the rejection of one’s own culture and focus only on the adoption of the outgroup culture, and separation is defined by the rejection of the outgroups’ culture and the motivation to maintain the culture of origin. Integration and assimilation are typically favored by majority and minority group members (Brown et al., 2016), and integration is positively associated with psychological and sociocultural adaptation (Abu-Rayya and Sam, 2016).

Berry’s acculturation model (1997) and extensions to or variations of his model, such as the interactive acculturation model (IAM, Bourhis et al., 1997), the concordance model of acculturation (CMA, Piontkowski et al., 2002) or the relative extended acculturation model (REAM, Navas et al., 2005) all respect the mutuality of acculturation by focusing on the bidimensionality and bidirectionality of acculturation processes between majority and minority members (Horenczyk et al., 2013). Consequently, intergroup processes between migrants and host society members shape acculturation orientations in both groups and also result from acculturation. Thus, for a better understanding of the acculturation process, it is important to identify variables that are capable of affecting both dimensions of acculturation orientations and also display the interdependence between attitudes and beliefs held by both groups, and to determine how they affect their behavior and how this behavior is evaluated by the outgroup. Several studies have supported a bidimensional approach to acculturation orientations (e.g., Ryder et al., 2000; Flannery et al., 2001) and have shown that the underlying dimensions may be interrelated rather than orthogonal (Zagefka et al., 2009; Van Acker and Vanbeselaere, 2011, 2012), and it has been recommended that both acculturation dimensions should be assessed separately (Rudmin and Ahmadzadeh, 2001; Rudmin, 2003; Brown and Zagefka, 2011; Van Acker and Vanbeselaere, 2011). In line with this research, we investigated the dimensions of cultural adoption and maintenance separately for refugees living in Germany.

Although theorizing on acculturation between majority and minority groups focuses on cultural adoption and maintenance motivations (Sam and Berry, 2016), it ignores the importance of experienced commonalities between the cultural groups in contact with each other. However, the acculturation process is characterized by permanent comparisons between the host society and immigrant groups in terms of cultural traditions and values, institutional practices, and everyday life. Thus, experiencing that an outgroup member feels the same way about cultural traditions, behaviors, or values builds up a shared reality containing cultural aspects of both groups and facilitating successful integration. Consequently, we suggest that research on acculturation orientations could benefit from additional variables that incorporate individual evaluations of matching aspects between one’s cultural ingroup and a relevant outgroup such as shared perspectives regarding education of children, basic democratic orientations, or work ethics. One concept that accounts for perceived commonalities between social groups is shared reality (Echterhoff et al., 2009; Echterhoff, 2012). Shared reality refers to an increased motivation of individuals to develop a common understanding with others about objects, people, social groups, or values. Thus, sharing implies that individuals experience their own attitudes toward an object of interest as converging with the attitudes of other individuals. Shared reality theory further suggests that the experience of commonalities with other individuals increases the motivation to build lasting relationships. Thus, the construction of a shared reality is not limited to interpersonal relationships. Conley et al. (2015) expanded shared reality theory to address intergroup relations between ethnic minority members and White Americans. They found that higher values in shared reality predicted less prejudice toward White Americans among African and Asian Americans as well as Latinos. In line with this research, we see shared reality as the belief that a person (as a social group member) perceives the world the same way as another group (Baldwin, 2001; Sinclair et al., 2006). The development of a shared reality may act as a bonding factor between the cultural ingroup and the host society that highlights cultural aspects, norms, or behaviors that both groups value. Therefore, it can work as a culturally collective closure (Dugas and Kruglanski, 2018) in building a social category that encompasses shared cultural aspects between majority and minority. Previous research has shown that shared reality of refugees with host society members increases due to positive contact experiences between both groups (Lutterbach and Beelmann, 2020).

### The Role of Stereotypes in Predicting Acculturation Orientations

The complex nature of acculturation processes is influenced by several psychological factors that shape majority and minority acculturation perspectives and thus cultural coexistence. Variables such as prejudice, social identity, similarity, and threat have been found to significantly affect acculturation-relevant orientations and beliefs (Zick et al., 2001; Florack et al., 2003; Zagefka et al., 2009, 2014; Brown and Zagefka, 2011; Navas et al., 2013; López-Rodríguez et al., 2014; López-Rodríguez and Zagefka, 2015). In addition, research has indicated that stereotypes also play a prominent role in predicting acculturation orientations (López-Rodríguez et al., 2014; López-Rodríguez and Zagefka, 2015). This is in line with the relative acculturation extended model (Navas et al., 2005) that recognizes the importance of psychosocial variables such as stereotypes in affecting majority and minority members’ acculturation perspectives. Furthermore, social cognition has been shown to significantly affect acculturation processes. Specifically, social learning and acquisition of knowledge about a new group (Rudmin, 2009) lead to changes in one’s stereotypical beliefs toward other social groups within the new cultural context (e.g., Stanciu and Vauclair, 2018; Stanciu et al., 2019).

In this context, stereotypes are beliefs about groups (Ashmore and Del Boca, 1981). Research on stereotypes differentiates between personal and consensual stereotypes (culturally shared beliefs about members of a distinct social group). Personal stereotypes are beliefs about social groups that have been found to be colored by personal experiences, motivational states, and individual differences (Jussim et al., 2015; Findor et al., 2020; Kotzur et al., 2020). In this study, we focused on the personal stereotypes held by the social group of refugees regarding German host society members. Research on stereotypes further distinguishes between a sociability and a competence dimension. This stems from the finding in research on person perception that trait ratings configure around intellectual versus social traits (Rosenberg et al., 1968). Subsequently, evidence around the stereotype content model (SCM, Fiske et al., 1999; Fiske et al., 2002) provided an empirical and a theoretical perspective on stereotypical judgments about social groups in terms of the categories “warmth” and “competence.” The sociability dimension measures beliefs on good or bad intentions of outgroup members and focuses on outgroup traits such as kindness and helpfulness (Leach et al., 2007; Brambilla et al., 2011, 2012). There is much evidence that the sociability dimension of stereotypes has a stronger impact on impression formation and outgroup evaluation compared to competence stereotypes (for an overview, see Abele and Wojciszke, 2014).

Research on the interrelation between stereotypes and acculturation orientations has found that positive sociability stereotypes correlate strongly and positively with cultural adoption motivations among majority group members (López-Rodríguez et al., 2014). Additionally, research by Alcott and Watt (2017) has indicated that the relation between sociability stereotypes and acculturation orientations among host society members is significantly more positive toward immigrants who are perceived as integrated or assimilated, whereas it is negative toward immigrants who are perceived as separated. However, this research is limited to stereotypes and acculturation orientations held by the cultural majority. The main contribution of the present study is to analyze the relation between stereotypes and acculturation orientations from the refugees’ point of view. Because the sociability dimension is associated more strongly with outgroup attitudes and acculturation relevant perceptions, we focused on the sociability dimension of stereotypes in the context of refugee migration, because it is primarily important to immigrants whether host societies will give assistance, peace, and shelter. Consequently, especially at the beginning of the acculturation process, stereotypes regarding whether or not host society members are friendly and helpful will be important to refugees. Furthermore, we analyzed the effects of both positive and negative sociability stereotypes held by refugees toward Germans on cultural adaptation, cultural maintenance, and shared reality to explore for varying effects of stereotype quality on different acculturation orientations.

### The Role of Perceived Discrimination

Acculturation orientations of majority and minority groups are not necessarily independent of each other (Arends-Tóth and van de Vijver, 2003; Matera et al., 2015), and power differences between the groups in contact with each other have a strong effect on the acculturation process (Sam and Berry, 2016). The majority constrains the choices of minority groups’ acculturation strategies by either openness to cultural diversity or endorsement of ethnocentrism and discrimination against immigrants. Thus, it is important to measure the acculturation climate produced by the host society through the eyes of minority group members. Experiences of discrimination against the cultural ingroup have been shown to significantly alter acculturation-relevant attitudes and orientations and affect the way migrants acculturate to a new society (Van Oudenhoven et al., 2006). Research on the interrelation between discrimination and acculturation perspectives has used both constructs as independent and dependent variables. There is correlational evidence that discrimination experiences negatively affect participation in host societies (Ramos et al., 2016); increase over time, slow down cultural adoption, and do not affect cultural maintenance orientations (Juang and Cookston, 2009); reduce the motivation for cultural maintenance (Bagci and Canpolat, 2019); and reduce host culture orientation (Kunst and Phillibert, 2018). In addition, a study by Jasinskaja-Lathi et al. (2003) found that different acculturation beliefs also change the way discrimination is experienced and interpreted. Regarding shared reality, two studies found that less perceived discrimination significantly predicted increases in minority group members’ perceptions of shared attitudes and values with majority group members (Conley et al., 2015; Lutterbach and Beelmann, 2020). The cited body of research typically uses one type of discrimination: everyday discrimination. This conceptualization of discrimination measures how regularly individuals perceive unfair treatment because of belonging to a socially devalued group. However, discrimination takes place in totally different social contexts and situations and can manifest in societal and governmental institutions. Thus, contextual discrimination focuses on unequal treatment of refugees by cultural, governmental, or societal institutions and services (e.g., by the police). Therefore, in the present study, we differentiated between everyday discrimination and contextual discrimination and analyzed their effects on acculturation orientations.

Despite the unique effects of stereotypes and discrimination, interactions between both variables might account for meaningful variations in acculturation orientations. Due to the bidimensionality and bidirectionality of acculturation (van Osch and Breugelmans, 2011; Horenczyk et al., 2013), refugees’ acculturation orientations depend on not only their intergroup stereotypical beliefs but also their intergroup experiences with host society members; and their acculturation orientations shape their intergroup attitudes and behaviors. Hence, it is important to analyze how variables interact that mirror social beliefs toward the cultural outgroup and experiences with the cultural outgroup in order to determine how they affect acculturation orientations.

### The Present Study

Whereas acculturation models and recent research suggest that stereotypes are important for the acculturation perspectives and preferences of majority group members, there has been little research on their role in the context of minority group members or even refugees. Thus, our present study aims to gather further empirical evidence on the interrelation between positive and negative sociability stereotypes and acculturation orientations from the refugees’ point of view. Furthermore, we add shared reality perceptions as a relevant acculturation orientation of refugees to differentiate the effects of refugees’ positive and negative sociability stereotypes toward German host society members on their motivation to adopt the host culture and to maintain their own culture. Second, due to the importance of mutuality in acculturation processes, we analyzed the major effect of refugees’ experiences of discrimination on acculturation orientations. Third, we provide first empirical evidence on how stereotypes and perceived discrimination interact in their effect on acculturation orientations from a refugee perspective, and in addition, we also differentiate between two types of discrimination (contextual and everyday discrimination) and analyze their diverse effects in combination with positive and negative sociability stereotypes.

We conducted the study in the context of German–refugee relations because of their social, political, and societal relevance in current German society in the aftermath of the so-called “refugee crisis” in 2015 and 2016. Since the start of this crisis, approximately 1.8 million refugees have migrated to Germany and applied for asylum (Statista, 2019) in order to find protection from war, civil unrest, persecution, or intolerable socioeconomic conditions. Most refugees migrated from middle eastern countries to Germany, and the largest minority groups in Germany due to forced migration are refugees from Syria (550,000), Afghanistan (220,000), and Iraq (180,000) (Statista, 2019).

#### Hypotheses

The literature on stereotypes and acculturation-relevant perspectives as well as research on the relationships between discrimination experiences and acculturation orientations suggest that both variables are capable of significantly affecting the acculturation process of refugees. To guide the analyses regarding the associations between positive and negative sociability stereotypes and contextual as well as everyday discrimination experiences with cultural adoption and maintenance motivations together with shared reality values among refugees in Germany, and to analyze interactions between stereotypes and discrimination experiences regarding acculturation variables, we tested the following hypotheses:

(1) Positive sociability stereotypes will be associated positively with cultural adoption and shared reality and negatively with cultural maintenance. Negative sociability stereotypes will relate negatively to cultural adoption and perceptions of a shared reality and positively to cultural maintenance.
(2) Experiences of contextual and everyday discrimination will be associated negatively with the motivation to adopt the host culture and perceptions of shared reality and positively with cultural maintenance orientations among refugees.
(3) Positive and negative sociability stereotypes and discrimination variables will elicit significant interactions with cultural adoption, cultural maintenance, and shared reality. Specifically, Hypothesis 3 tests 12 interactions in total—that is, four interactions (positive sociability stereotypes × contextual discrimination, positive sociability stereotypes × everyday discrimination; negative sociability stereotypes × contextual discrimination, negative sociability stereotypes × everyday discrimination) on all three dependent variables. In general, we expect that that the negative association between discrimination and cultural adoption as well as shared reality and the positive association between discrimination and cultural maintenance will become more intense in the case of higher values in positive sociability stereotypes and less intense in the case of higher values in negative sociability stereotypes.

## Materials and Methods

### Participants

The final refugee sample consisted of 783 refugees in Thuringia, Germany. We excluded 123 participants from the original dataset, because of substantial amounts of missing values on the study variables (more than 50 percent). The remaining sample had some missing data (less than 3 percent) that was imputed via linear interpolation. Regarding sociodemographic variables (see Table 1), data indicated 71.5 percent male and 27.8 percent female participation (five individuals reported no gender); the age of participants ranged between 18 and 68 years (*M* = 31.64, *SD* = 10.39); and nationality consisted of 49.2 percent Syrian, 27.0 percent Afghan, 13.5 percent Iraqi, and 5.0 percent Iranian (remaining 5.3 percent migrated mainly from North African countries to Germany). Furthermore, refugees reported their educational level (19.0 percent stated no graduation at all, 15.8 percent with an elementary school degree, 19.8 percent completed middle school, 29.9 percent graduated from high school, and 15.4 percent reported a university degree), the length of their stay in Germany (6.9 percent migrated in 2013/14, 73.2 percent in 2015/16, and 19.9 percent in 2017/18), their residency status (with 57.5 percent having a residency permit), the context in Germany (86.8 percent urban vs. 13.2 percent rural) and their religious affiliation (64.6 percent Sunni, 17.9 percent Shiite, 5.9 percent Christian, 6.9 percent other, and 4.6 percent reporting no religion). Regarding gender, age, and variation in origin of participants, the dataset matched the distribution of refugees in Thuringia at the time data were collected (Bundesamt für Migration und Flüchtlinge BAMF, 2019).

**TABLE 1:** Demographic variables of the total sample of refugees and the three largest refugee subgroups.

	**Total sample**	**Afghan refugees**	**Iraqi refugees**	**Syrian refugees**
**Variables**	***n* = 783**	***n* = 210**	***n* = 105**	***n* = 383**
**Gender**				
Male	71.5	84.1	71.4	66.8
Female	27.8	15.9	28.6	33.2
Age	31.64(10.39)	28.54(9.23)	33.50(11.21)	32.70(10.72)
**Religion**				
Sunni	64.6	35.7	40.9	89.7
Shiite	17.9	51.9	15.5	0.7
Christian	5.9	6.4	2.7	3.0
Other	6.4	2.6	36.4	2.1
None	4.6	3.4	4.5	4.4
**Context**				
Urban	86.6	83.2	86.7	89.8
Rural	13.2	16.8	13.3	10.2
Residency status				
Residency permit	57.5	42.9	50.5	72.6
Other	42.5	57.1	49.5	27.4
**Length of stay**				
2013/2014	6.9	8.6	1.0	7.6
2015/2016	73.2	85.2	68.6	77.8
2017/2018	19.9	6.2	30.5	14.6
**Education**				
No graduation	19.0	46.4	18.1	7.6
Elementary school	15.8	20.8	14.3	13.4
Middle school	19.8	12.1	22.9	23.8
High school	29.9	15.5	16.7	37.4
University	15.4	5.3	18.1	17.8

*All demographic variables in percent except for age (in means and standard deviations).*

### Procedure and Measures

Data were collected as part of the project *Thüringen Monitor Integration* (Beelmann et al., 2019) that surveyed refugees in urban and rural areas of Thuringia, Germany in 2018 (data were collected during the first half of 2018). The questionnaire was translated into an Arabic and a Persian version via back-translation (Peña, 2007). Trained native speakers (mostly students from Friedrich Schiller University with a refugee migration background themselves) gathered the data in sheltered accommodations for refugees, refugee associations, language and integration courses, mosques, at the Friedrich Schiller University Jena, and in private settings. Participation was limited to refugees who migrated to Germany between 2013 and 2018 and had a minimum age of 18 years. In the beginning, refugees were informed about the purpose of the study and how to respond to the questionnaire. Any questions they had were answered by the native speakers. Participants received a compensation of 10 euro after completing the questionnaire. On average, refugees took between 90 and 120 min to complete the questionnaire. Alongside the following measures (see Table 2 and Appendix), the questionnaire contained further subsections that asked for flight experiences and actual situation in Germany, integration-relevant political and social attitudes, contact experiences and friendships with Germans, language skills and integration courses, as well as future expectations.

**TABLE 2:** Correlations between the study variables and descriptive statistics.

**Variable**	***M(SD)***	**2**	**3**	**4**	**5**	**6**	**7**
(1) Positive sociability stereotypes	3.40(0.79)	–0.25**	–0.31**	–0.16**	0.50**	–0.05	0.59**
(2) Negative sociability stereotypes	2.45(0.75)	–	0.25**	0.33**	–0.19**	–0.03	–0.21**
(3) Contextual discrimination	1.59(0.50)		–	0.43**	–0.14**	0.05	–0.34**
(4) Everyday discrimination	1.62(0.43)			–	–0.11**	–0.03	–0.26**
(5) Cultural adoption	4.05(0.72)				–	–0.20**	0.63**
(6) Cultural maintenance	3.77(0.87)					–	–0.13*
(7) Shared reality	3.46(0.99)						–

Both types of discrimination were measured on three-point scales and the remaining study variables on a five-point rating scale. *p < 0.05; **p < 0.01.

Stereotypes were operationalized in line with the sociability dimension of stereotype content (Brambilla and Leach, 2014). We differentiated between a positive and a negative sociability dimension regarding German host society members and assessed each quality from the refugee perspective with three items on a five-point rating scale asking to what extent stereotypes apply to all Germans in general from a refugee group perspective. Positive sociability stereotypes were assessed with the adjectives *gentle*, *helpful*, and *trustworthy* whereas negative sociability stereotypes were measured with the adjectives *arrogant*, *hostile*, and *rejecting*. The rating ranged from 1 = *nobody* to 5 = *all*. Higher values indicated more positive and negative sociability stereotypes. The internal consistency of the positive sociability scale was α = 0.76 and reached α = 0.66 in case of the negative sociability scale.

Acculturation orientations were assessed with two nine-item scales (Nguyen and Von Eye, 2002; Berry et al., 2006) measuring the motivation to adopt the German host society culture or maintain one’s own cultural identity in the German context. The cultural adaptation scale asked refugees to imagine future life in Germany and how likely it was that they would adopt German traditions, values, and behaviors (e.g., *When I think about my future life in Germany, I would like to adopt German values*). On the contrary, the cultural maintenance scale assessed the same items regarding the motivation to retain one’s cultural traditions, values, and behaviors when living in Germany (e.g., *When I think about my future life in Germany, I would like to maintain the values of my country of origin*). Both scales measured acculturation orientations on a five-point scale from 1 = *disagree completely* to 5 = *agree completely*. Higher values indicated higher motivation for cultural adaptation and maintenance. Internal consistency was α = 0.83 for cultural adoption and α = 0.88 for cultural maintenance.

Shared reality perceptions were operationalized with three items asking how far refugees thought their attitudes, experiences, and perspectives on everyday life match those held by Germans (Conley et al., 2015; Lutterbach and Beelmann, 2020). The scale ranged from 1 = *disagree completely* to 5 = *agree completely*. Refugees rated, for example, the item: *Germans and I share the same outlook on the world*. Higher values indicated increased shared reality. The internal consistency of the scale was α = 0.77.

Discrimination experiences were measured with two scales. First, contextual discrimination was assessed in terms of discriminatory experiences by refugees in different contexts such as *institutional* contexts, by the *police*, or when *looking for a new apartment* (Worbs et al., 2016). This instrument measured discrimination with eight items on a three-point scale ranging from 1 = *no experience at all* to 3 = *very often*. The internal consistency of the scale was α = 0.85. Second, we applied the *Everyday Discrimination Scale* (Clark et al., 2004; Lewis et al., 2012) to differentiate between different types of discrimination against refugees via four items (e.g., *I was offended by Germans*). Again, everyday discrimination was measured on a three-point scale from 1 = *no experience at all* to 3 = *very often*. Internal consistency was α = 0.77. Higher values indicated more perceived contextual as well as everyday discrimination.

## Results

Table 2 reports means (*M*), standard deviations (*SD*), and zero-order correlations of all measures. Refugees reported having more positive sociability stereotypes (*M* = 3.40, *SD* = 0.79) than negative sociability stereotypes (*M* = 2.45, *SD* = 0.75) toward German host society members, *t*(782) = 122.74, *p* < 0.001, and experienced the same amount of contextual (*M* = 1.59, *SD* = 0.50) as well as everyday discrimination (*M* = 1.62, *SD* = 0.43) caused by Germans, *t*(782) = −1.12, *p* = 0.262. The correlations between positive sociability stereotypes and cultural adoption (*r* = 0.50, *p* = 0.002) and shared reality (*r* = 0.59, *p* = 0.001) were significant and positive, and, in contrast, significantly negative between negative sociability stereotypes and cultural adoption (*r* = −0.19, *p* = 0.006) and shared reality values (*r* = −0.21, *p* = 0.005). Regarding the correlations between the discrimination scales and acculturation variables, contextual discrimination correlated significantly and negatively with cultural adoption (*r* = −0.14, *p* = 0.007); and shared reality (*r* = −0.34, *p* = 0.004) and everyday discrimination also correlated significantly negatively with cultural adoption (*r* = −0.11, *p* = 0.008) and shared reality (*r* = −0.26, *p* = 0.005). Only cultural adoption (*r* = −0.20, *p* = 0.005) and shared reality values (*r* = −0.13, *p* = 0.024) correlated significantly and negatively with cultural maintenance values.

We conducted a hierarchical regression analysis to test the unique effects of positive and negative sociability stereotypes (Hypothesis 1) and contextual as well as everyday discrimination (Hypothesis 2) on cultural adaption, cultural maintenance, and shared reality. Additionally, we tested for interaction effects between the stereotypes and discrimination variables in predicting acculturation orientations (Hypothesis 3). Thus, in Step 1 we entered the demographic variables gender, age, religion, context, residency status, length of stay, education, and social group as predictors of cultural adoption, cultural maintenance, and shared reality. In Step 2, we entered positive and negative sociability stereotypes in addition to the sociodemographic variables. Step 3 analyzed the effects of contextual and everyday discrimination on the three acculturation outcomes, controlling for demographic factors. In Step 4, we entered positive and negative sociability stereotypes as well as contextual and everyday discrimination experiences and demographic variables as predictors of acculturation orientations (to analyze the simple effects regarding the interaction effects). Finally, in Step 5, we added interaction terms between the stereotype and discrimination variables to analyze the interaction effects on cultural adoption, cultural maintenance and shared reality. For the interaction analysis in Step 5, we mean-centered the variables measuring stereotypes and discrimination (Field, 2013). A *post hoc* sensitivity analysis revealed that the *R*^2^ increase from Step 4 to Step 5 (inclusion of four interaction terms leading to an *R*^2^ increase of 0.01 in all dependent variables due to a total of 25 predictor variables) had a power of 0.77 for data from 783 refugees (calculated with G^∗^Power 3.1) in the case of cultural adaption and shared reality. Regarding cultural maintenance, the sensitivity analysis showed a power of 0.67. Consequently, analyses had sufficient power for the detection of a small effect regarding the prediction of cultural adoption and shared reality, but was limited in the case of cultural maintenance (Cohen, 1988).

Table 3 reports the hierarchical regression analysis. Regressions weights in Step 1 revealed that cultural adoption was predicted significantly and negatively by gender (β = −0.11, *p* = 0.001), predicted positively by age, predicted positively by a Christian in reference to a Sunni religious affiliation, and predicted negatively by refugees who reported no education or middle school education compared to refugees with a high school degree. Regarding cultural maintenance, increased motivations to hold on to refugees’ ingroup culture were predicted positively by gender and negatively by length of stay (in comparison to refugees migrated in 2015/2016), and predicted negatively by Afghan and Iraqi refugees in reference to Syrian refugees. Shared reality was predicted positively by age, predicted negatively by no education and middle school education compared to refugees with high school education, and predicted negatively by Afghan in reference to Syrian refugees. Demographic variables accounted for a significant amount of variance in cultural adoption, *R*^2^ = 0.08, *F*(*df* = 8, 775) = 9.04, *p* < 0.001, cultural maintenance, *R*^2^ = 0.03, *F*(*df* = 8, 775) = 4.59, *p* < 0.001, and shared reality, *R*^2^ = 0.11, *F*(*df* = 8, 775) = 12.09, *p* < 0.001.

**TABLE 3:** Hierarchical regression analysis predicting acculturation orientations by demographic variables, sociability stereotypes, and discrimination experiences as well as interactions between sociability stereotypes and discrimination.

	**Cultural adoption**				**Cultural maintenance**				**Shared reality**			
**Model**	***B***	***SE***	**β**	***p***	***B***	***SE***	**β**	***p***	***B***	***SE***	**β**	***p***
**1 (Constant)**	3.77	0.16		<0.001	4.13	0.19		<.001	3.33	0.20		<0.001
Gender (men = 0; women = 1)	–0.19	0.05	–.13	<0.001	0.19	0.07	0.10	0.004	–0.11	0.08	–0.05	0.186
Age	0.01	0.01	0.11	0.001	–0.01	0.01	–0.02	0.488	0.01	0.01	0.14	<0.001
**Religion (Reference = Sunni)**												
Shiite – Sunni	–0.04	0.07	–0.06	0.517	–0.08	0.08	–0.09	0.346	0.03	0.10	0.03	0.749
Christian – Sunni	0.24	0.11	0.35	0.030	–0.08	0.14	–0.10	0.550	0.10	0.16	0.10	0.530
Other – Sunni	–0.12	0.13	–0.18	0.352	0.01	0.16	0.01	0.990	–0.23	0.19	–0.23	0.241
None – Sunni	0.05	0.11	0.08	0.623	0.09	0.14	0.11	0.494	0.18	0.16	0.18	0.276
Context (rural = 0; urban = 1)	0.07	0.07	0.03	0.325	–0.02	0.09	–0.01	0.812	0.16	0.10	0.06	0.122
Residency status (permit = 0; other = 1)	–0.04	0.05	–0.03	0.455	–0.02	0.07	–0.01	0.773	–0.15	0.08	–0.08	0.056
**Length of stay (Reference = 2015, 2016)**												
2013/2014 – 2015/2016	–0.05	0.10	–0.08	0.578	–0.25	0.12	–0.30	0.035	–0.11	0.14	–0.11	0.443
2017/2018 – 2015/2016	0.06	0.07	0.09	0.382	–0.24	0.09	–0.29	0.006	0.014	0.11	0.14	0.188
**Education (Reference = High school)**												
No graduation – high school	–0.20	0.08	–.28	0.016	0.10	0.10	0.11	0.316	–0.34	0.11	–0.35	0.003
Elementary school – high school	–0.12	0.07	–0.17	0.118	0.09	0.09	0.11	0.338	–0.16	0.11	–0.17	0.138
Middle school – high school	–0.20	0.07	–0.29	0.004	–0.01	0.08	–0.02	0.866	–0.24	0.10	–0.25	0.015
University – high school	–0.03	0.08	–0.04	0.690	–0.18	0.09	–0.21	0.052	–0.05	0.11	–0.05	0.621
**Social group (Reference Syrian refugees)**												
Afghanistan – Syria	–0.04	0.07	–0.05	0.593	–0.24	0.08	–0.34	<0.001	–0.34	0.10	–0.35	<0.001
Iraq – Syria	0.14	0.07	0.21	0.054	–0.18	0.09	–0.25	<0.001	0.10	0.11	0.10	0.359

**2 (Constant)**	2.98	0.19		<0.001	4.19	0.26		<0.001	2.06	0.27		<0.001
Gender (men = 0; women = 1)	–0.17	0.05	–0.11	<0.001	0.19	0.07	0.10	0.004	–0.09	0.07	–0.04	0.213
Age	0.01	0.01	0.06	0.036	–0.01	0.01	–0.02	0.507	0.01	0.01	0.09	0.004
**Religion (Reference = Sunni)**												
Shiite – Sunni	–0.09	0.06	–0.14	0.123	–0.08	0.08	–0.09	0.357	–0.05	0.09	–0.05	0.586
Christian – Sunni	0.19	0.10	0.29	0.050	–0.08	0.14	–0.09	0.558	0.03	0.14	0.03	0.842
Other – Sunni	–0.15	0.12	–.23	0.193	0.01	0.16	0.01	0.983	–0.27	0.17	–0.27	0.112
None – Sunni	0.05	0.10	0.06	0.646	0.10	0.14	0.11	0.488	0.18	0.14	0.18	0.207
Context (rural = 0; urban = 1)	0.09	0.06	0.04	0.138	–0.02	0.09	–0.01	0.811	0.21	0.09	0.07	0.025
Residency status (permit = 0; other = 1)	–0.04	0.05	–0.02	0.470	–0.02	0.07	–0.01	0.770	–0.14	0.07	–0.07	0.040
**Length of stay (Reference = 2015, 2016)**												
2013/2014 – 2015/2016	–0.06	0.09	–0.09	0.479	–0.25	0.12	–0.29	0.036	–0.11	0.12	–0.11	0.379
2017/2018 – 2015/2016	0.03	0.07	0.03	0.687	–0.25	0.09	–0.29	0.006	0.08	0.09	0.08	0.406
**Education (Reference = High school)**												
No graduation – high school	–0.18	0.07	–0.26	0.012	0.10	0.10	0.11	0.318	–0.32	0.10	–0.32	0.002
Elementary school – high school	–0.11	0.07	–0.16	0.112	0.09	0.09	0.11	0.338	–0.14	0.10	–0.14	0.150
Middle school – high school	–0.17	0.06	–0.25	0.007	–0.02	0.08	–0.02	0.855	–0.20	0.09	–0.20	0.028
University – high school	–0.01	0.07	–0.01	0.907	–0.18	0.09	–0.21	0.051	0.01	0.10	0.01	0.921
Social group (Reference Syrian refugees)												
Afghanistan – Syria	–0.03	0.06	–0.05	0.588	–0.24	0.09	–0.34	<0.001	–0.24	0.09	–0.24	0.009
Iraq – Syria	0.17	0.07	0.25	0.010	–0.19	0.09	–0.25	<0.001	0.14	0.10	0.15	0.123
**Positive sociability stereotypes**	**0.35**	**0.03**	**0.41**	**<0.001**	**–0.01**	**0.04**	**–0.01**	**0.723**	**0.52**	**0.04**	**0.41**	**<0.001**
**Negative sociability stereotypes**	**–0.08**	**0.03**	**–0.09**	**0.005**	**–0.02**	**0.04**	**–0.01**	**0.970**	**–0.19**	**0.04**	**–0.14**	**<0.001**

**3 (Constant)**	4.41	0.19		<0.001	3.94	0.24		<0.001	4.44	0.27		<0.001
Gender (men = 0; women = 1)	–0.21	0.05	–0.14	<0.001	0.20	0.07	0.10	0.003	–0.16	0.07	–0.08	0.035
Age	0.01	0.01	0.09	0.010	–0.01	0.01	–0.01	0.592	0.01	0.01	0.11	0.002
**Religion (Reference = Sunni)**												
Shiite – Sunni	–0.05	0.07	–0.06	0.502	–0.07	0.08	–0.08	0.375	0.01	0.10	0.01	0.924
Christian – Sunni	0.19	0.11	0.27	0.089	–0.06	0.14	–0.07	0.642	–0.01	0.16	–0.01	0.983
Other – Sunni	–0.12	0.12	–0.18	0.348	0.01	0.16	0.01	0.999	–0.22	0.19	–0.22	0.248
None – Sunni	0.04	0.11	0.05	0.728	0.10	0.14	0.11	0.460	0.13	0.16	0.14	0.396
Context (rural = 0; urban = 1)	0.06	0.07	0.03	0.403	–0.02	0.09	–0.01	0.845	0.14	0.11	0.05	0.169
Residency status (permit = 0; other = 1)	–0.05	0.05	–0.03	0.375	–0.02	0.07	–0.01	0.788	–0.16	0.08	–0.08	0.038
**Length of stay (Reference = 2015, 2016)**												
2013/2014 – 2015/2016	–0.06	0.09	–0.07	0.594	–0.025	0.12	–0.29	0.034	–0.11	0.14	–0.11	0.439
2017/2018 – 2015/2016	0.03	0.07	0.09	0.387	–0.024	0.09	–0.28	0.007	0.13	0.10	0.13	0.208
**Education (Reference = High school)**												
No graduation – high school	–0.20	0.08	–0.30	0.010	0.11	0.10	0.12	0.272	–0.39	0.11	–0.40	<0.001
Elementary school – high school	–0.12	0.07	–0.18	0.101	0.09	0.09	0.10	0.334	–0.17	0.11	–0.17	0.116
Middle school – high school	–0.22	0.07	–0.32	0.001	–0.06	0.08	–0.01	0.945	–0.29	0.10	–0.30	0.003
University – high school	–0.04	0.07	–0.06	0.588	–0.18	0.09	–0.02	0.057	–0.07	0.11	–0.08	0.483
Social group (Reference Syrian refugees)												
Afghanistan – Syria	0.02	0.07	0.02	0.790	–0.26	0.09	–0.35	<0.001	–0.24	0.10	–0.24	0.018
Iraq – Syria	0.14	0.07	0.20	0.062	–0.18	0.09	–0.24	<0.001	0.07	0.11	0.08	0.479
**Contextual discrimination**	**–0.21**	**0.06**	**–0.15**	**<0.001**	**–0.11**	**0.07**	**–0.05**	**0.049**	**–0.31**	**0.08**	**–0.16**	**<0.001**
**Everyday discrimination**	**–0.04**	**0.06**	**–0.02**	**0.497**	**0.13**	**0.08**	**0.06**	**0.045**	**–0.30**	**0.09**	**–0.13**	**<0.001**

**4 (Constant)**	3.10	0.22		<0.001	3.98	0.30		<0.001	2.69	0.31		<0.001
Gender (men = 0; women = 1)	–0.19	0.05	–0.13	<0.001	0.20	0.07	0.10	0.003	–0.12	0.07	–0.06	0.083
Age	0.01	0.01	0.07	0.044	–0.01	0.01	–0.01	0.585	0.01	0.01	0.08	0.010
**Religion (Reference = Sunni)**												
Shiite – Sunni	–0.09	0.06	–0.13	0.498	–0.08	0.08	–0.08	0.373	–0.05	0.09	–0.05	0.545
Christian – Sunni	0.17	0.10	0.27	0.090	–0.06	0.14	–0.07	0.644	–0.03	0.14	–0.03	0.859
Other – Sunni	–0.16	0.12	–0.23	0.351	0.01	0.16	0.01	0.996	–0.26	0.17	–0.27	0.118
None – Sunni	0.04	0.06	0.04	0.713	0.11	0.14	0.12	0.445	0.15	0.14	0.15	0.289
Context (rural = 0; urban = 1)	0.09	0.09	0.04	0.169	–0.02	0.09	–0.01	0.862	0.19	0.09	0.07	0.039
Residency status (permit = 0; other = 1)	–0.04	0.07	–0.03	0.410	–0.02	0.07	–0.01	0.786	–0.15	0.07	–0.07	0.031
**Length of stay (Reference = 2015,2016)**												
2013/2014 – 2015/2016	–0.06	0.09	–0.09	0.486	–0.25	0.12	–0.29	0.036	–0.12	0.12	–0.11	0.366
2017/2018 – 2015/2016	0.03	0.07	0.04	0.649	–0.25	0.09	–0.28	0.006	0.08	0.09	0.08	0.397
**Education (Reference = High school)**												
No graduation – high school	–0.17	0.07	–0.26	0.015	0.11	0.10	0.12	0.271	–0.35	0.10	–0.35	<0.001
Elementary school – high school	–0.11	0.07	–0.17	0.097	0.09	0.09	0.10	0.327	–0.15	0.10	–0.15	0.127
Middle school – high school	–0.17	0.06	–0.26	0.005	–0.01	0.09	–0.01	0.945	–0.23	0.09	–0.23	0.011
University – high school	–0.01	0.07	–0.01	0.976	–0.17	0.09	–0.20	0.059	–0.01	0.10	–0.01	0.926
**Social group (Reference Syrian refugees)**												
Afghanistan – Syria	0.06	0.06	0.09	0.360	–0.26	0.10	–0.34	<0.001	–0.18	0.09	–0.18	0.043
Iraq – Syria	0.17	0.07	0.26	0.009	–0.18	0.09	–0.24	<0.001	0.13	0.10	0.13	0.171
**Positive sociability stereotypes**	**0.35**	**0.03**	**0.41**	<**0.001**	–**0.01**	**0.04**	–**0.01**	**0.899**	**0.49**	**0.04**	**0.39**	<**0.001**
**Negative sociability stereotypes**	–**0.07**	**0.03**	–**0.08**	**0.017**	–**0.01**	**0.04**	–**0.01**	**0.780**	–**0.16**	**0.04**	–**0.11**	<**0.001**
**Contextual discrimination**	–**0.12**	**0.05**	–**0.09**	**0.015**	**0.05**	**0.07**	**0.03**	**0.451**	–**0.18**	**0.07**	–**0.09**	**0.012**
**Everyday discrimination**	**0.06**	**0.06**	**0.04**	**0.306**	**0.06**	**0.08**	**0.02**	**0.456**	–**0.16**	**0.08**	–**0.07**	**0.049**

**5 (Constant)**	3.07	0.22		<0.001	4.01	0.31		<0.001	2.65	0.32		<0.001
Gender (men = 0; women = 1)	–0.19	0.04	–0.13	<0.001	0.20	0.07	0.11	0.003	–0.12	0.07	–0.06	0.084
Age	0.01	0.01	0.07	0.033	–0.01	0.01	–0.01	0.642	0.01	0.01	0.09	0.008
**Religion (Reference = Sunni)**												
Shiite – Sunni	–0.08	0.06	–0.12	0.196	–0.06	0.08	–0.07	0.457	–0.05	0.09	–0.05	0.583
Christian – Sunni	0.18	0.10	0.27	0.067	–0.05	0.14	–0.06	0.692	–0.01	0.14	–0.01	0.923
Other – Sunni	–0.18	0.12	–0.26	0.137	–0.05	0.16	–0.06	0.763	–0.29	0.17	–0.29	0.088
None – Sunni	0.03	0.10	0.04	0.795	0.10	0.14	0.12	0.471	0.14	0.14	0.14	0.333
Context (rural = 0; urban = 1)	0.09	0.06	0.05	0.154	–0.01	0.09	–0.01	0.888	0.20	0.09	0.07	0.032
Residency status (permit = 0; other = 1)	–0.04	0.05	–0.02	0.389	–0.02	0.07	–0.01	0.787	–0.14	0.07	–0.07	0.043
**Length of stay (Reference = 2015/2016)**												
2013/2014 – 2015/2016	–0.06	0.09	–0.08	0.524	–0.24	0.12	–0.28	0.044	–0.10	0.12	–0.11	0.402
2017/2018 – 2015/2016	0.02	0.07	0.03	0.794	–0.26	0.09	–0.30	0.004	0.07	0.09	0.07	0.434
**Education (Reference = High school)**												
No graduation – high school	–0.17	0.07	–0.26	0.013	0.10	0.10	0.12	0.289	–0.35	0.10	–0.36	<0.001
Elementary school – high school	–0.12	0.07	–0.18	0.083	0.09	0.09	0.11	0.328	–0.15	0.10	–0.15	0.121
Middle school – high school	–0.17	0.06	–0.26	0.005	–0.01	0.08	–0.01	0.963	–0.23	0.09	–0.23	0.010
University – high school	0.01	0.07	0.01	0.949	–0.16	0.09	–0.19	0.075	0.01	0.10	0.01	0.961
**Social group (Reference Syrian refugees)**												
Afghanistan – Syria	0.06	0.06	0.09	0.336	–0.24	0.09	–0.34	<0.001	–0.17	0.09	–0.17	0.059
Iraq – Syria	0.17	0.07	0.27	0.007	–0.17	0.09	–0.23	<0.001	0.14	0.10	0.14	0.137
Positive sociability stereotypes	0.02	0.03	0.02	0.682	–0.59	0.04	–0.37	<0.001	0.49	0.04	0.39	<0.001
Negative sociability stereotypes	–0.23	0.04	–0.24	<0.001	–0.16	0.05	–0.09	<0.001	–0.15	0.05	–0.11	0.001
Contextual discrimination	–0.36	0.05	–0.22	<0.001	–0.98	0.07	–0.38	<0.001	–0.19	0.07	–0.09	0.012
Everyday discrimination	–0.71	0.05	–0.37	<0.001	–0.47	0.08	–0.37	<0.001	–0.17	0.08	–0.07	0.037
**Positive sociability stereotypes × Contextual discrimination**	**0.13**	**0.01**	**0.29**	<**0.001**	**0.23**	**0.02**	**0.37**	<**0.001**	**0.08**	**0.02**	**0.13**	<**0.001**
**Positive sociability stereotypes × Everyday discrimination**	**0.11**	**0.01**	**0.22**	<**0.001**	**0.17**	**0.02**	**0.20**	<**0.001**	**0.19**	**0.02**	**0.33**	<**0.001**
**Negative sociability stereotypes × Contextual discrimination**	–**0.06**	**0.01**	–**0.11**	<**0.001**	**0.03**	**0.02**	**0.04**	**0.068**	**0.01**	**0.02**	**0.01**	**0.648**
**Negative sociability stereotypes × Everyday discrimination**	**0.14**	**0.01**	**0.33**	<**0.001**	**0.05**	**0.02**	**0.08**	<**0.001**	**0.02**	**0.02**	**0.03**	**0.376**

*Bold variables display simple effects and interactions of stereotypes and discrimination on acculturation orientations and shared reality.*

In testing Hypothesis 1, Step 2 of the hierarchical regression showed that cultural adoption was predicted significantly and positively by positive sociability stereotypes as well as being predicted significantly and negatively by negative sociability stereotypes. Cultural maintenance orientations were not predicted by either quality of sociability stereotypes. However, shared reality was predicted positively by positive sociability stereotypes and negatively by negative sociability stereotypes. Demographic and stereotype variables accounted for an increased and significant amount of explained variance in cultural adoption, *R*^2^ = 0.27, *F*(*df* = 19, 764) = 12.60, *p* < 0.001, Δ*R*^2^ = 0.19, shared reality, ^*R*2^ = 0.30, *F*(*df* = 19, 764) = 4.54, *p* < 0.001, Δ*R*^2^ = 0.19, and cultural maintenance, ^*R*2^ = 0.15, *F*(*df* = 19, 764) = 6.06, *p* = 0.001, Δ*R*^2^ = 0.01.

Step 3 of the hierarchical regression tested Hypothesis 2 by analyzing the unique effect of contextual and everyday discrimination experiences on acculturative orientations held by refugees living in Germany. Cultural adoption was predicted significantly and negatively by contextual discrimination but not by everyday discrimination. The orientation to maintain one’s culture in the host context was predicted negatively by contextual discrimination and predicted positively by everyday discrimination. Regarding shared reality values, contextual as well as everyday discrimination were significant and negative predictors. In contrast to Step 1, demographic and discrimination variables accounted for an increased and significant amount of explained variance in cultural adoption, ^*R*2^ = 0.11, *F*(*df* = 19, 764) = 4.14, *p* < 0.001, Δ*R*^2^ = 0.03, cultural maintenance, ^*R*2^ = 0.16, *F*(*df* = 19, 764) = 6.15, *p* < 0.001, Δ*R*^2^ = 0.01, and shared reality, ^*R*2^ = 0.16, *F*(*df* = 19, 764) = 6.45, *p* < 0.001, Δ*R*^2^ = 0.05.

To test Hypothesis 3, we analyzed the simple effects of both stereotype measures and discrimination variables on cultural adoption, cultural maintenance, and shared reality (Step 4), which, in turn, delivered the basis to interpret the interaction effects resulting from Step 5.

Thus, Step 4 integrated positive as well as negative sociability stereotypes and contextual as well as everyday discrimination to predict acculturation orientations, and it tested for interaction recommendations in regressions (Field, 2013). Cultural adoption was predicted significantly and positively by positive sociability stereotypes and predicted negatively by negative sociability stereotypes and contextual discrimination. Cultural maintenance was not predicted significantly by both sociability stereotype variables or by both discrimination experiences. Regarding shared reality, positive sociability stereotypes appeared as a positive predictor, and negative sociability stereotypes as well as contextual and everyday discrimination were found as negative predictors. Demographic, stereotype, and discrimination variables increased the amount of significant explained variance in cultural adoption, *R*^2^ = 0.28, *F*(*df* = 21, 762) = 11.90, *p* < 0.001, Δ*R*^2^ = 0.01 compared to Step 2, and Δ*R*^2^ = 0.16 compared to Step 3, cultural maintenance, *R*^2^ = 0.16, *F*(*df* = 21, 762) = 5.60, *p* < 0.001, Δ*R*^2^ = 0.01 in comparison to Step 2 (no increment in variance explanation in comparison to Step 3), and shared reality, *R*^2^ = 0.32, *F*(*df* = 21, 762) = 14.70, *p* < 0.001, Δ*R*^2^ = 0.02 regarding Step 2, and Δ*R*^2^ = 0.16 concerning Step 3.

**FIGURE 1:**
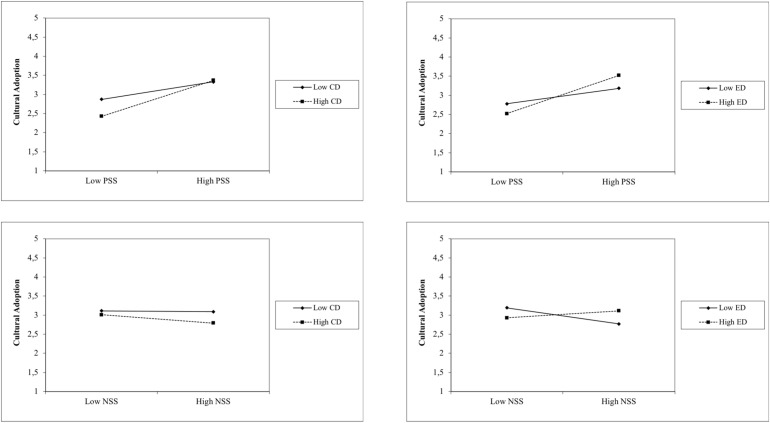
Interactions between sociability stereptypes and discrimination experiences among refugees on cultural adoption orientations. PSS, positive sociability stereotypes; NSS, negative sociability stereotypes; CD, contextual discrimination; ED, everyday discrimination.

Step 5 analyzed 12 interactions between sociability stereotypes and discrimination experiences on acculturation orientations held by refugees in Germany. Regarding cultural adoption, all interaction terms elicited significant effects. Besides the negative interaction term between negative sociability stereotypes and contextual discrimination, the interaction effects between positive sociability stereotypes and contextual as well as everyday discrimination and between negative sociability stereotypes and everyday discrimination were all positive (see Figure 1). Regarding the simple effects, the positive interaction terms in the case of positive sociability stereotypes indicated that with a stronger negative association between contextual as well as everyday discrimination and cultural adoption, the positive relation between positive sociability stereotypes and cultural adoption became more powerful. The positive interaction between negative sociability stereotypes and everyday discrimination showed that with an intensified negative relation between negative sociability stereotypes and cultural adoption, the positive relation between everyday discrimination and cultural adoption became stronger. Furthermore, the negative interaction term between negative sociability stereotypes and contextual suggested that a stronger negative association between negative sociability stereotypes and adoption motivations was accompanied by a less negative relation between contextual discrimination and cultural adoption (or vice versa).

**FIGURE 2:**
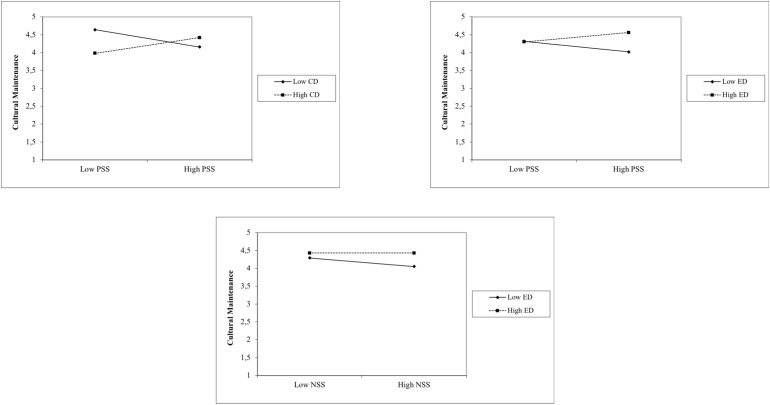
Cross-over interactions between sociability stereptypes and discrimination experiences among refugees on cultural maintenance orientations. PSS, positive sociability stereotypes; NSS, negative sociability stereotypes; CD, contextual discrimination; ED, everyday discrimination.

The interaction analysis of cultural maintenance showed three significant positive interactions between (a) positive sociability stereotypes and contextual discrimination, (b) positive sociability stereotypes and everyday discrimination, and (c) negative sociability stereotypes and everyday discrimination. This indicated crossover interactions (Aiken and West, 1991) due to the non-significant main effects of positive and negative sociability stereotypes and contextual and everyday discrimination on cultural maintenance orientations (see Step 4). Figure 2 shows these crossover effects, indicating that refugees with higher levels in positive sociability stereotypes and both discrimination variables reported a stronger motivation to maintain their culture. In contrast, refugees with high values in negative sociability stereotypes and low experiences of everyday discrimination were found to report a decreased motivation to retain their ingroup’s culture.

**FIGURE 3:**
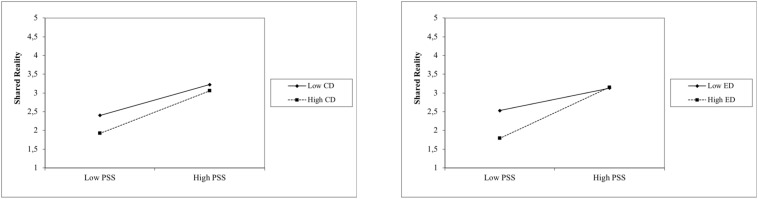
Interactions between sociability stereptypes and discrimination experiences among refugees on shared reality. PSS, positive sociability stereotypes; CD, contextual discrimination; ED, everyday discrimination.

Finally, shared reality was predicted significantly by positive interactions between positive sociability stereotypes and contextual as well as everyday discrimination (see Figure 3). Regarding the simple effects resulting from Step 4, the positive interactions terms of positive sociability stereotypes indicated that with a stronger negative association between contextual as well as everyday discrimination and shared reality, the positive relation between positive sociability stereotypes and shared reality became more intense.

In comparison to Step 4, demographic, stereotype, discrimination, and interaction variables accounted for small and significant increments of explained variance in cultural adoption, *R*^2^ = 0.29, *F*(*df* = 25, 758) = 10.80, *p* < 0.001, Δ*R*^2^ = 0.01, cultural maintenance, *R*^2^ = 0.17, *F*(*df* = 25, 758) = 5.23, *p* < 0.001, Δ*R*^2^ = 0.01, and shared reality, *R*^2^ = 0.33, *F*(*df* = 25, 767) = 12.80, *p* < 0.001, Δ*R*^2^ = 0.01.

With regard to the significant impact of different demographic indicators on all acculturation orientations, we additionally conducted interaction analyses between the stereotype, discrimination, and demographic variables. In the case of gender, there were two significant interactions. Negative sociability stereotypes and gender interacted positively on cultural adoption (β = 0.29, *p* < 0.001) and shared reality (β = 0.37, *p* < 0.001) indicating that the negative effect of negative sociability stereotypes (see Table 3, Step 2, β = −0.09, *p* < 0.001, in case of cultural adoption, β = −0.14, *p* = 0.004, in case of shared reality) was stronger among female refugees (simple effects for females were β = −0.11, *p* < 0.001 in case of cultural adoption, and β = −0.04, *p* < 0.001, in case of shared reality). All other interaction effects were not significant. We also calculated three-way interactions to test for demographic interference regarding the 12 interactions between stereotypes and discrimination experiences, but no significant interaction terms emerged.

## Discussion

The present study examined how positive and negative sociability stereotypes toward host society members and contextual as well as everyday discrimination experiences influenced the acculturation orientations and shared reality perceptions held by refugees. It also analyzed interactions between stereotypes and discrimination experiences regarding the impact on acculturation orientations and shared reality values.

### Unique Effects of Sociability Stereotypes and Discrimination Experiences

Regarding Hypothesis 1, results revealed that the perception of host society members as sociable was associated positively with the motivation of refugees to adopt relevant German cultural traditions and values as well as to perceive commonalities with the German host culture. Nevertheless, these positive sociability stereotypes held by refugees were not associated significantly with the motivation to maintain their cultural identity when imagining their future life in Germany. Negative sociability stereotypes toward German host society members were related negatively to the motivation to adopt the German host culture, and also associated negatively with shared reality values; but as in the case of positive sociability stereotypes, there was no significant association between negative sociability stereotypes and cultural maintenance orientations. Thus, Hypothesis 1 was supported regarding the asymmetrical associations between positive and negative sociability stereotypes and cultural adoption motivations as well as shared reality, but not in the case of cultural maintenance orientations.

These findings confirm that positive sociability stereotypes are fundamentally related to acculturation orientations and shared reality perceptions among refugees, and they are in line with previous research on the relationship between stereotypes and acculturation preferences among majority group members (Lee and Fiske, 2006; Maisonneuve and Testé, 2007). For example, the studies by Montreuil and Bourhis (2001, 2004) found that acculturation preferences in the majority point of view differ regarding the valuation or devaluation of immigrant groups. The study by López-Rodríguez et al. (2014) also showed that majority stereotypes were associated with acculturation preferences for immigrants. Furthermore, the research by López-Rodríguez et al. (2014) found that the interrelation between stereotypes and cultural adoption was stronger than the association with cultural maintenance—a result also found by Maisonneuve and Testé (2007). However, the asymmetrical effects of positive and negative sociability stereotypes on cultural adoption orientations are problematic. Successful integration is defined by both cultural adaption and cultural maintenance (Berry, 1997, 2001), but negative sociability stereotypes are accompanied by decreases in the motivation to adopt aspects of the host society. Thus, this pattern suggests that negative sociability stereotypes may promote the acculturation strategy of separation, although it is rarely preferred by either majority or minority groups (Brown et al., 2016).

Another important aim of the current study was to examine the impact of different discrimination experiences on refugees’ acculturation orientations to respect acculturative experiences with the host society. We examined contextual and everyday discrimination experiences. Regarding Hypothesis 2, results mostly indicated that both discrimination subtypes were associated negatively with cultural adoption and shared reality values. However, in the case of cultural maintenance orientations, contextual discrimination related negatively to the motivation to maintain one’s culture, and in contrast, everyday discrimination experiences related positively to higher values in cultural maintenance.

Like other studies on the relationship between discrimination experiences and acculturation orientations (Berry et al., 2006; Te Lindert et al., 2008; Juang and Cookston, 2009), the results of our analyses point in the direction that perceived discrimination is associated with lower acculturation into the host society. This is also stressed by longitudinal evidence reported by Ramos et al. (2016) showing that perceived discrimination is associated with a perceived reduction of permeability, which, in turn, results in avoiding the host society, and simultaneously endorsing one’s own cultural group. Nonetheless, other longitudinal research has indicated that the link between both concepts is stronger, when discrimination is predicted by acculturation orientations (Motti-Stefanidi et al., 2018). Furthermore, our study highlights that everyday discrimination leads to a stronger motivation to maintain one’s cultural heritage. This is in line with the rejection–identification model (Branscombe et al., 1999) stating that minority group members focus more strongly on their cultural ingroup to seek the protection of ingroup members against discrimination by the majority. But interestingly, contextual discrimination works the other way around by reducing the motivation to maintain cultural aspects of the ingroup. Discriminatory and prejudiced contexts are locations and situations with predictable and systematic inequalities that are longstanding, invariable, and highly dependent on social group membership (Murphy et al., 2018). Thus, refugees might be motivated to discard aspects of their cultural ingroup in favor of ending systematic experiences of discrimination.

### Interactions Between Sociability Stereotypes and Discrimination Experiences

The most important contribution of this article is the novel evidence on the interaction between stereotypes and discrimination experiences, and how these interactions are associated with acculturation orientations among refugees. We found substantial support for our Hypothesis 3, because nine of the 12 interaction terms were associated significantly with acculturation orientations. Overall, the pattern of results emphasized, as expected, that discrimination experiences of refugees become more intensified when refugees hold high values in positive sociability stereotypes, and become less important when refugees already hold high values in negative sociability stereotypes toward host society members.

Regarding cultural adoption orientations and shared reality values, interactions between positive sociability stereotypes and both types of discrimination resulted in a disillusion effect. Because the interactions were positive, a stronger positive relation between positive sociability stereotypes and cultural adoption as well as shared reality was associated with stronger negative relations between discrimination experiences and both dependent measures. Thus, it is especially refugees who are likely and motivated to adopt aspects of the host culture and to perceive commonalities with host society members due to their positive sociability stereotypes who suffer from discrimination by the hosting society regarding their motivation to adopt the host culture and to develop a shared reality. In contrast, the interaction between negative sociability stereotypes and contextual discrimination showed that a stronger negative relationship between negative stereotypes and cultural adoption was associated with a weaker negative relation between contextual discrimination and cultural adoption or vice versa. This interaction indicated that with a stronger rejection of the host society culture due to negative beliefs about host society members, discrimination experiences become less meaningful for refugees’ cultural adoption orientations and shared reality values.

Regarding cultural maintenance, analyses revealed crossover interactions (Aiken and West, 1991). Thus, there were no significant associations between cultural maintenance orientations and positive and negative sociability stereotypes, and contextual and everyday discrimination. However, crossover interactions indicate that the association between a predictor and a dependent variable is opposite, depending on the value of the second predictor. Hence, the interaction between positive sociability stereotypes and contextual as well as everyday discrimination indicated that refugees with high values in positive sociability stereotypes and high values in perceived discrimination reported a pronounced motivation to maintain their own culture. Furthermore, the interaction between negative sociability stereotypes and everyday discrimination showed that high levels in everyday discrimination experiences were associated with higher levels in cultural maintenance among low and high levels of negative stereotypes.

Taken together, our research gives ample evidence that strong discrimination experiences elicit a maladaptive effect on successful integration, because they reduce the motivation to adopt aspects of the host culture, reduce the perception of sharedness between the host society and one’s cultural ingroup, and increase the motivation to maintain one’s own culture among refugees holding strong positive sociability stereotypes toward the host society. Hence, increased negative encounters and discrimination experiences are likely to lead to separating acculturation strategies among refugees who actually had the potential to integrate into the host society.

Research on intergroup contact has shown that individuals who are high in positive outgroup evaluations, high in their motivation to learn about social outgroups, and highly motivated to extend their social self tend to seek more intergroup encounters (Kauff et al., 2020). Consequently, refugees with positive sociability stereotypes and an increased motivation to adopt cultural aspects of the host society end up in more intergroup experiences, situations, and contexts. Thus, increased intergroup contact increases the potential for experiencing discrimination. Such disillusioning experiences within the acculturation process are likely to lead to acculturative stress (Berry, 2006), especially in the case of an experienced ambiguity between one’s own motivations toward biculturality and perceptions that host society members are not motivated to accept other cultures.

### Limitations and Strengths of the Present Research

The cross-sectional nature of the study is a major impediment to causal inferences concerning the bidirectionality of acculturation. Future research should assess stereotypes, discrimination, and acculturation orientations among majority and minority groups in a longitudinal approach to fully analyze and understand the mutuality of acculturation between migrants and their host society.

In addition, generalizability is limited by the significant impact of various sociodemographic variables on the dependent acculturation measures. However, the sample was representative for refugees living in Germany in terms of gender, age, and country of origin (Beelmann et al., 2019). Concerning the educational level, the sample was much more educated than the average refugee population that migrated to Germany. Furthermore, interaction analyses between stereotypes and demographics as well as between discrimination experiences and demographics as well as three-way interactions found only three significant interactions regarding gender and residency status. Thus, our main analyses were mostly free of covariate interactions.

Regarding the assessment of stereotypes, our research is limited to personal stereotypes. Consensual or cultural stereotypes have been found to be more accurate, less positive, and less colored by individual experiences, motivational states, and individual differences (Jussim et al., 2015; Findor et al., 2020; Kotzur et al., 2020). Furthermore, cultural stereotypes are likely to be more connected with real positions of social groups within societal structures.

From a measurement point of view, some of our measures could be improved and extended. We used only three items to measure both positive and negative sociability stereotypes. Nonetheless, the effects of stereotypes on acculturation orientations replicate most findings of earlier research on the interrelation between stereotypes and acculturation in a majority sample (López-Rodríguez et al., 2014). Hence, we do not think these limited measures had any major consequences in terms of threatening the validity of our results. Regarding potential new measures, it would be necessary to develop an instrument measuring all dimensions of shared reality and not just perceived commonalities between ingroup and outgroup. A comprehensive measure of shared reality has to consist of a cognitive dimension regarding contents to share, an affective dimension regarding the experience of commonalities with outgroup members, and a metadimension regarding the sensation that the outgroup member experiences this commonality as well.

Another limitation stems from the translation of the items into Arabic and Persian. The data acquisition used the translation technique of back-translation by native speakers as well as the method of decentering (Sechrest et al., 1972) to ensure linguistic equivalence (Peña, 2007). However, specific linguistic concepts and constructs cannot be translated into another cultural context without describing or defining their meanings and contents (e.g., terms from German migration law that have no equivalent in Arabic or Persian). This is especially problematic in the case of paper-and-pencil questionnaires. Nevertheless, data collection was always accompanied by native speakers of both Persian and Arabic language groups to explain the modus operandi of the questionnaire and to answer questions raised by the participants.

Regarding the strengths of the present research, the study is the first to analyze the relationships between positive and negative sociability stereotypes and acculturation orientations as well as shared reality from a refugee perspective. Furthermore, the analyses tested for interaction effects between stereotypes and discrimination to respect the bidimensionality and bidirectionality of the acculturation process. In addition, the methodological strengths of this study are that it is based on a large sample of refugees living in Thuringia, Germany, there were a series of demographic variables to control for the hierarchical regression analysis, multiitem scales of the analyzed variables, and mostly enough power to detect small interaction effects.

### Implications for Future Research and Practice

Future research should investigate the interrelation between stereotypes and acculturation orientations among both refugees and host society members and contrast the effects of sociability but also of competence and morality stereotypes on preferences for cultural adoption, cultural maintenance, and the development of a shared understanding with the cultural outgroup. Furthermore, future empirical research should contrast the effects of different dimensions of stereotypes regarding the social ingroup and the social outgroup among minority and majority groups in predicting acculturative orientations. Another line of research might look for variables that promote successful integration of refugees into the host society to determine whether such variables are capable of increasing motivation to adopt the host culture and maintain the culture of one’s origin. Regarding the interaction between stereotypes and discrimination, it might be interesting to add other variables such as threat to identity (Molina et al., 2015; Tsukamoto and Fiske, 2017), realistic or symbolic threat (Stephan and Renfro, 2002), empathy (Maisonneuve and Taillandier-Schmitt, 2016), or other important intergroup variables that could interact with stereotypes or discrimination experiences in predicting acculturation motivations by both majority and minority group members. These variables might also act as protective factors to reduce the maladaptive power of discrimination experiences on successful integration.

Regarding practice, the present results indicate that programs designed to promote successful acculturation should address majority group member prejudices and tendencies to discriminate against refugees (Beelmann and Lutterbach, 2020). Promoting refugees’ social skills so that they can deal more competently with the stereotypes and discriminatory behavior of the host society could be an additional strategy to support integration efforts (e.g., Beelmann et al., 2020). In general, these programs need to be designed in a culturally sensitive way (Castro et al., 2010; Sundell et al., 2016) that reflects the target-group-specific stereotypes and acculturation motivations while making people aware of the impact of perceived discrimination on acculturation orientations. The challenge is to come up with ways to construct such programs so that they do not further intensify tensions between both cultural groups, but promote the creation of integration-relevant attitudes and the recognition that discrimination plays a role in social life and should not lead to maladaptive acculturation strategies such as separation or marginalization.

## Conclusion

The main contribution of this study was to bring together stereotypes toward host society members held by refugees and perceptions of discrimination provoked by host society members to analyze intergroup beliefs and experiences and their effects on acculturation-relevant orientations. Alongside the unique effects of positive and negative stereotypes, and contextual as well as everyday discrimination on acculturation perceptions of refugees, we explored interactions between these variables. Discrimination was maladaptive, especially among refugees high in positive sociability stereotypes, resulting in a stronger rejection of the host culture and a pronounced motivation to maintain one’s own culture in the new cultural context.

To ensure successful acculturation between refugees and host society members, public and political debates, policy, and integration practices have to focus on and discuss the problem of discrimination against refugees. Societal strategies need to reduce systemic discrimination, but also develop a cultural climate that promotes diversity and multiculturality. Facilitating positive encounters between refugees and host society members sets a foundation for reduced negative stereotypes, reduced discrimination, and reduced threat in both groups, and it enables acculturation strategies that are characterized by adopting the outgroup culture and maintaining one’s own cultural identity—that is, successful integration.

## Data Availability Statement

The dataset presented in this article was uploaded to GESIS. Requests to access the dataset should be directed to https://www.gesis.org/angebot/archivieren-und-registrieren/datenarchivierung/datenzugang/.

## Ethics Statement

The studies involving human participants were reviewed and approved by Ethics Center at Friedrich Schiller University. The patients/participants provided their written informed consent to participate in this study.

## Author Contributions

SL: data aquisition, execution of data analysis, and text preparation. AB: project management and text preparation. Both authors contributed to the article and approved the submitted version.

## Conflict of Interest

The authors declare that the research was conducted in the absence of any commercial or financial relationships that could be construed as a potential conflict of interest.

## Appendix: Items

### Cultural Adoption

When I think about my future life in Germany, I would like to adopt German values.

When I think about my future life in Germany, I would like to adopt German traditions.

When I think about my future life in Germany, I would like to adopt the German language.

When I think about my future life in Germany, it is important to me to socialize with Germans.

When I think about my future life in Germany, I would like to adopt German gender conventions.

When I think about my future life in Germany, I would like to have German friends.

When I think about my future life in Germany, I would like to adopt German behaviors.

When I think about my future life in Germany, I would like to adopt German values regarding child education.

When I think about my future life in Germany, I would like to adopt German work values.

### Cultural Maintenance

When I think about my future life in Germany, I would like to maintain the values of my country of origin.

When I think about my future life in Germany, I would like to maintain the traditions of my country of origin.

When I think about my future life in Germany, I would like to maintain the language of my country of origin.

When I think about my future life in Germany, it is important to me to socialize with people from my country of origin.

When I think about my future life in Germany, I would like to maintain gender conventions in line with the conventions of my country of origin.

When I think about my future life in Germany, I would like to have friends from my country of origin.

When I think about my future life in Germany, I would like to maintain behaviors that are typical for my country of origin.

When I think about my future life in Germany, I would like to maintain values regarding child education.

When I think about my future life in Germany, I would like to maintain work values that are typical for my country of origin.

### Shared Reality

Germans and I share the same outlook on the world.

My attitudes are quite similar to those held by most Germans.

If I were to interact with a German person, chances are good that we would agree about lots of things.

### Positive and Negative Sociability Stereotypes

Regarding the group of Germans in general, for how many Germans do the following characteristics apply?*Positive: Gentle, helpful, trustworthy. Negative: arrogant, hostile, rejecting.*

### Contextual Discrimination

To what extent have you been disadvantage in Germany, because of being a refugee?*Regarding job search; by state institutions; at a restaurant; while looking for an apartment; at the hospital; by the police; at the club; in public transportation.*

### Everyday Discrimination

To what extent have you made negative experiences in your everyday life, because of being a refugee in Germany?*I was treated without respect.*

I was offended by Germans.

I was threatened by Germans.

I was physically attacked by Germans.
